# Exploration of circulating metabolites in infants with abusive head trauma

**DOI:** 10.1016/j.crneur.2025.100154

**Published:** 2025-06-28

**Authors:** Estelle Maret, Tatjana Sajic, Kim Wiskott, Sylvain Le Gludic, Federica Gilardi, Youssef Daali, Tony Fracasso, Aurélien Thomas

**Affiliations:** a Faculty Unit of Toxicology, University Center of Legal Medicine, Lausanne University Hospital, Chemin de la Vulliette 4, 1000 Lausanne 25, Switzerland; b Unit of Forensic Toxicology and Chemistry, University Center of Legal Medicine, Lausanne University Hospital and Geneva University Hospital, Lausanne, Geneva, Switzerland; c Unit of Forensic Medicine, University Center of Legal Medicine, Rue Michel-Servet 1, 1211 Geneva 4, Switzerland; d Department of Anesthesiology, Pharmacology, Intensive Care and Emergencies, Faculty of Medicine UNIGE, Rue Michel Servet 1, 1211 Geneva 14, Switzerland

**Keywords:** Pediatric abusive head trauma, Serum metabolomics

## Abstract

Abusive head trauma (AHT) is a severe form of traumatic brain injury (TBI) and causes significant brain lesions by vigorous shaking. It is the leading cause of mortality and morbidity in children under 2 years of age. If not fatal, AHT can result in severe disabilities, often requiring long-term care. Clinical diagnosis of AHT is challenging, because symptoms are often non-specific, overlap with those of other diseases and relies on screening for intracranial, spinal, and ocular lesions. To date, no screening test has been developed to preselect children suspected to be victims of AHT for further clinical investigations. However, as recently demonstrated via analysis of serum proteomes of infant victims of AHT, large-scale omic analysis of blood serum samples could help identify molecules with high potential for early detection of human pathologies. Here, we investigated the circulating serum metabolome of infants with severe head trauma with a Glasgow Coma Scale (GCS) score of 3–4 and compared it to infants with no signs of head trauma during medico-legal examinations. Using liquid chromatography coupled to high resolution mass spectrometry (LC-HRMS), we identified 53 metabolites with the most significant differences between the groups. Six metabolites were already known to be implicated in different gross pathologies associated with neurological diseases. In addition, our analysis revealed several lipids and lipid-like molecules, all with an increased profile in the peripheral blood circulation of infant victims of AHT. As we speculated some of the identified metabolites to come from specific brain regions affected by the shaking mechanism, we further performed a multi-omic integration by integrating metabolites showing evidence of their presence in the brain and publicly available proteomic data. As results, we found significant metabolite-protein correlations which could be closely associated with AHT, thus, providing evidence of tensions and supporting strong dynamic changes occurring within the brain during assault.

## Highlights

• Distinct peripheral blood metabolome in Abusive Head Trauma (AHT) infants.
• Some identified metabolites are tightly associated with neurological diseases.
• Some metabolites might originate from specific and susceptible brain regions.
• Metabolites and proteins of specific brain regions show similar variations.
• Proof-of principle metabolomics study of blood serum of infant victims of AHT.

## Graphical abstract

**Figure:**
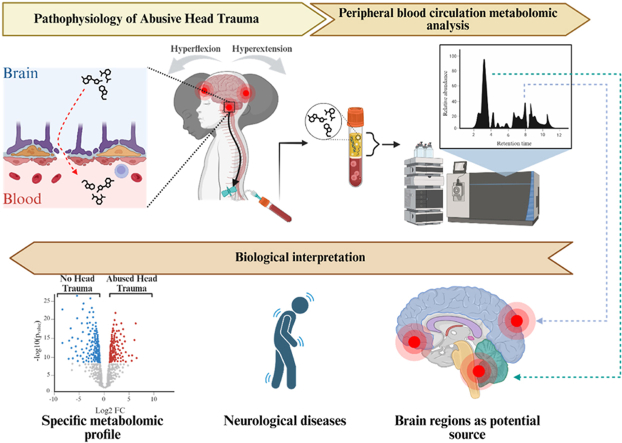

## Abbreviations

14(S)-HDHA: 14(S)-hydroxy docosahexaenoic acid
5-HIAA: 5-hydroxyindole-3-acetic acid
AA: Arachidonic acid
AHT: Abusive head trauma
ANPEP: Aminopeptidase N
APOE: Apolipoprotein E
AST: Astrocytes
BBB: Blood-brain barrier
BG: Basal ganglia
CB: Cerebellum
CD: Choroid
CFHR1: Complement factor H-related protein 1
CLU: Clusterin
COMP: Cartilage oligomeric matrix protein
COLEC10: Collectin-10
CP: Ceruloplasmin
CPC: Cone photoreceptor cells
CT: Cerebral cortex
EXN: Excitatory neurons
F5: Coagulation factor V
FC: Fold-change
FDR: False discovery rate
GCS: Glasgow Coma Scale
GP1BA: Platelet glycoprotein Ib alpha chain
GPX3: Glutathione peroxidase 3
HABP2: Hyaluronan-binding protein 2
HC: Hippocampus
HP: Haptoglobin
HMDB: Human metabolome database
HT: Hypothalamus
IGF2: Insulin-like growth factor II
IGFBP2: Insulin-like growth factor-binding protein 2
IGHV3-33: Immunoglobulin heavy variable 3-33
IGHV3-48: Immunoglobulin heavy variable 3-48
INN: Inhibitory neurons
JCHAIN: Immunoglobulin J chain
LBP: Lipopolysaccharide-binding protein
LC-MS: Liquid chromatography mass spectrometry
LC-MS/MS: Liquid chromatography coupled to tandem mass spectrometry
MAN2A1: Alpha-mannosidase 2
MASP1: Mannan-binding lectin serine protease 1
MB: Midbrain
MCGC: Microglial cells
MD: Medulla
MGC: Muller glia cells
MS: Mass spectrometry
NEG: Negative ion mode
OB: Olfactory bulb
OIT3: Oncoprotein-induced transcript 3 protein
OLC: Oligodendrocyte cells
OLPC: Oligodendrocytes precursor cells
PA: Pimelic acid
PO: Pons
POS: Positive ion mode
PRM: Parallel reaction monitoring
PZP: Pregnancy zone protein
PROS1: Vitamin K-dependent protein S
RARRES2: Retinoic acid receptor responder protein 2
RPC: Rod photoreceptor cells
S1P: Erytho-sphingosine 1-phosphate
SAA1: Serum amyloid A-1 protein
SC: Spinal cord
SCC: Schwann cells
SERPIND1: Heparin cofactor 2
SIDS: Sudden infant death syndrome
TBI: Traumatic brain injury
TL: Thalamus
ULCMS: Ultra-liquid chromatography mass spectrometry grade

## 1 Introduction

Abusive head trauma (AHT) in young infants is one of the most severe forms of traumatic brain injury (TBI) and is the leading cause of mortality and morbidity in children under two years of age. Vigorous shaking of infants causes significant brain injury (Minns et al., 2012; Fujiwara et al., 2008; Myhre et al., 2007; Tung et al., 2006; Keenan et al., 2004; Bechtel et al., 2004), with subdural hematoma, retinal hemorrhage and encephalopathy being the most common occurring injuries. Non-specific symptoms of AHT are easily attributed to accidental head injury, viral illness, feeding difficulties, or colic (Vinchon et al., 2010; Jenny et al., 1999; Letson et al., 2016). The lack of a well-established screening test to identify children at risk of abuse contributes to the high incidence of misdiagnosis. A previous study has shown that one third of infants are misdiagnosed on their first visit to the physician, requiring three visits for a correct diagnosis (Thorpe et al., 2014), thus delaying treatment and reducing chances of the survival. Mortality and morbidity of these infants are linked to a range of outcomes, from mild learning disabilities to severe handicaps and premature death (Hung, 2020; Mian et al., 2015).

As brain trauma induces the permeability of the blood-brain barrier (BBB) and axonal shearing (Jarrahi et al., 2020; Kaur and Sharma, 2018), brain components associated with brain injury can be detected in the bloodstream (Morochovič et al., 2009; Žurek and Fedora, 2012; Arun et al., 2012), leading to potential source of TBI biomarkers. Some efforts have been invested on the development of early detection of AHT with proteomic studies, notably with the validation of multivariable model for intracranial hemorrhage in infants (Berger et al., 2005, 2006a, 2006b, 2009, 2017) and high-throughput protein screening of peripheral blood circulation of AHT, with the recent highlight of 4 brain-tissue specific proteins (Wiskott et al., 2023). Regarding metabolomic investigations following TBI, adults serum showed the increase of metabolites in the bloodstream potentially indicating disruption of BBB, while neuroprotectant metabolites showed decreased profile indicating their potential transport to the injured brain (Orešič et al., 2016). It has also been demonstrated in a postnatal rats study that decreased levels of cardiolipins in the brain following TBI significantly correlated with their increase in plasma (Anthonymuthu et al., 2019). Therefore, these interesting findings showed metabolomics as a promising tool for TBI investigation.

Here, we explored the peripheral blood metabolome in infants who suffered severe head injuries due to AHT and compared their metabolomes with those who died of sudden infant death syndrome (SIDS). We used LC-HRMS and identified metabolites with particular interest in AHT. We further investigated our metabolomic results with literature review, a publicly available metabolome atlas of the aging mouse brain (Ding et al., 2021) and performed metabolome data integration with published AHT proteomic data (Wiskott et al., 2022). We sought to identify metabolites that may have a potential impact on the pathophysiology of AHT in children and could lead to more in-depth research in the field of head trauma.

## 2 Methods

### 2.1 AHT and atraumatic control samples

Antemortem peripheral blood circulation samples were collected in additive-free tubes between 2013 and 2018, then frozen at −80 °C until analysis. The Research Ethics Committee of Geneva, Switzerland (Project ID, 2021-01304) approved for the reuse of these samples (art. 34 of the Swiss Human Research Act) in our retrospective study. Our study involved two distinct groups: AHT cases (n = 4) previously evaluated according to the guidelines of the French High Authority of Health (Haute Autorité de Santé, 2017) and atraumatic controls (infants died of SIDS, n = 4) evaluated according to The San Diego classification, which applies when the cause of death remains unexplained after a thorough investigation (Bajanowski et al., 2006) (Supplementary Table 1). Both samples of AHT and SIDS groups were processed identically. Among the AHT cases, only one infant exhibited peripheral injury, specifically ecchymoses (bruising) on the left upper limb. In contrast, none of the SIDS cases showed any evidence of head trauma.

### 2.2 Metabolomic study

#### 2.2.1 Sample preparation

The extraction solution was prepared with 5 μl of phenobarbital-d5 and 5 μl of hydrocodone-d6. Solution was completed up to 5 ml of MeOH ultra liquid chromatography mass spectrometry grade (ULCMS)/EtOH (50:50, v/v). 200 μl of extraction solution were added to 50 μl of serum samples. Solutions were vortexed and then centrifugated for 20 min at 13,000 g at 4 °C. Supernatants were kept and dry evaporated with nitrogen with Caliper TurboVap® LV Evaporator (Marshall Scientific) at room temperature. Dry samples were reconstituted with 50 μl of H_2_O/MeOH ULCMS (90:10, v/v). Solutions were vortexed and centrifuged for 20 min at 13,000 g at 4 °C. Solutions were transferred into injection vials to be injected. A QC solution was prepared by pooling 5 μl of each sample, injected at the beginning and at the end of the analysis and used for assessment of analytical precision.

#### 2.2.2 Untargeted metabolomic analysis by liquid chromatography-high resolution mass spectrometry (LC-HRMS) and data acquisition

Samples were analyzed using an Ultimate 3000 with C18 Kinetex, 50 × 2.1 mm 2.6 μm + KrudKatcher ULTRA HPLC in-line filter 2.0 μm depth filter × 0.004 in ID and Orbitrap Exploris 120 operating in positive and negative mode. 5 μl of H_2_O/MeOH ULCMS (90:10, v/v), QC solution and samples were injected at 45 °C with a flow of 0.3 ml/min for HPLC. Eluants were (A) H_2_O ULCMS + 0.1 % formic acid and (B) MeOH ULCMS + 0.1 % formic acid. The gradient program was set as follows: 2 % B (0–0.3 min), 2 %–98 % B (0.3–6 min), 98 %–100 % B (6–9 min), 100 %–2 % B (9–9.1 min) and 2 % B (9.1–13 min). LC-HRMS configuration was set with Xcalibur™ Software (Thermo Fisher Scientific™) and TraceFinder software (Thermo Fisher Scientific™ - version 5.1). Orbitrap resolution was 120,000 with 70–1,000 (m/z) as scan range. Solutions were then stored at −80 °C for further analysis.

#### 2.2.3 LC-HRMS data processing and statistical analysis

Positive (POS) and negative (NEG) LC-HRMS data were analyzed and visualized with MS-DIAL software (version 4.9.221218). The parameters used in MS-DIAL were set as follows: data type: centroid with 0,01 Da mass tolerance, ionization type: hard ionization, minimum peak height: 10,000 amplitude, sigma window value: 0.5, mass range: 0–2000 Da, alignment: 0.05 min retention time and 0.015 Da mass tolerance with 75 % peak detected in at least one group.

Data were log_10_ transformed using RStudio software (version 2022.02.3 + 492). Principal component analysis (PCA) scores of the two principal components of all features for both ion modes (13,515 in NEG and 35,589 in POS) were generated with RStudio software (version 2022.02.3 + 492) using “factoextra” package and used to assess the analytical precision (Supplementary Fig. 1).

Features were then filtered based on m/z with HMDB all metabolite data (Version 5.0, released on 2021-11-17), with adduct types set by default with M + H and M-H depending on POS and NEG mode. Difference mass allowed at 5 ppm between matching the two sets of data. Features matching HMDB database were kept for downstream analysis.

The Student's t-test with false discovery rate (FDR) was used as the strategy for the selection of features for subsequent identification, based on the calculation of statistically different changes between the groups. FDR corrected p-value (q-value) was considered significant when FDR ≤0.05. Then, features showing at least 10-time fold-changes differences between groups (Student's t-test, FDR ≤0.05; FC ≤ −10 and FC ≥ 10; AHT *vs.* SIDS) were selected and subject to fragmentation and identification using LC-HRMS/MS technology. RStudio software was used for statistical computing (version 2022.02.3 + 492).

#### 2.2.4 Targeted metabolomic analysis by liquid chromatography-high resolution mass spectrometry (LC-HRMS/MS) and parallel reaction monitoring (PRM) analysis

Before injection into LC-HRMS/MS, samples were pooled by groups. H_2_O/MeOH ULCMS (90:10, v/v), QC solution and pooled samples were injected in Ultimate 3000 with C18 Kinetex, 50 × 2.1 mm 2.6 μm + KrudKatcher ULTRA HPLC in-line filter 2.0 μm depth filter × 0.004 in ID and Orbitrap Exploris 120 in positive and negative modes. 5 μl of solutions were injected at 45 °C with a flow of 0.3 ml/min. Eluants were (A) H_2_O ULCMS + 0.1 % formic acid and (B) MeOH ULCMS + 0.1 % formic acid. The gradient program was set as follows: 2 % B (0–0.3 min), 2 %–98 % B (0.3–6 min), 98 %–100 % B (6–9 min), 100 %–2 % B (9–9.1 min) and 2 % B (9.1–13 min). LC-HRMS/MS configuration was set with Xcalibur™ software (Thermo Fisher Scientific ™) and TraceFinder software (Thermo Fisher Scientific ™ - version 5.1). Orbitrap resolution was 60,000 with 70–1,000 (m/z) as scan range. A whole spectral scan was performed with fragmentation at targeted mass and retention time window with a mass tolerance at ± 10 ppm. Dynamic exclusion of fragmentation was set after 1 time, and 1 s. Intensities threshold was set at 2 × 10^5^. Data dependent scan properties were set with 2 m/z isolation window, 30 % HCD collision energies and orbitrap resolution at 15,000.

MzCloud™ advanced mass spectral database (HighChem LLC) was used for metabolites identification. PRM analysis was then assessed: m/z and RT of each identified metabolite was evaluated for matching its respective untargeted feature, with maximum difference tolerance criteria of 5.00 ppm for m/z and ±0.3 min for RT. Peaks were then checked with Compound Discoverer™ and identified metabolites were attributed to their respective feature.

#### 2.2.5 Over-representation analyses (ORA)

Features from LC-HRMS analysis showing significant differences between groups (FC ≤ −10 and FC ≥ 10, Student's t-test, FDR ≤0.05) were enriched based on their mass according to HMDB. ORA was performed with MetaboAnalyst 5.0 online using enrichment analysis tool (https://www.metaboanalyst.ca/MetaboAnalyst/home.xhtml, accessed August 14th, 2023) and enriched features were compared to gross pathologies (Supplementary Table 2). Gross pathologies with enrichment ratio ≥0.3 and p-value ≤0.1 were kept for biological analysis and further supported by identified metabolites. Graphics were visualized with Microsoft Excel and prism software (version 9).

#### 2.2.6 Brain-related metabolites

Brain region specificity for metabolomic analysis were reported from the metabolome atlas of aging mice brain (Ding et al., 2021) (Supplementary Table 3). RStudio software was used for statistical computing (version 2022.02.3 + 492) and graphics were visualized with prism software (version 9). Student's t-test was used, and FDR corrected p-value was considered significant when FDR ≤0.05. Spearman's correlation between each metabolite was set significant when rho = 0.7, df = 6.

### 2.3 Integration analysis

Available proteomics raw files were downloaded from Wiskott et al., 2023) study via ProteomeXchange (Perez-Riverol et al., 2021). The 165 significantly dysregulated proteins (two-sided Student's t-test, P < 0.05 and Cohen's d > 1.56, power 0.8) were filtered with two sets of transcriptomic lists. Proteins with gene expression with mention “elevated in brain” and “elevated in glial and/or neuronal cell populations” from the Human Protein Atlas database (Supplementary Tables 3 and 4) were selected. Proteins were named by their gene name from UniProt database. R software was used for statistical computing and graphics visualization (version 2022.02.3 + 492). From their respective samples, Spearman's correlation was computed between proteomic and metabolomic data and set significant when rho = 0.7, df = 6.

## 3 Results

### 3.1 Outline of the metabolomic workflow

**Fig. 1:**
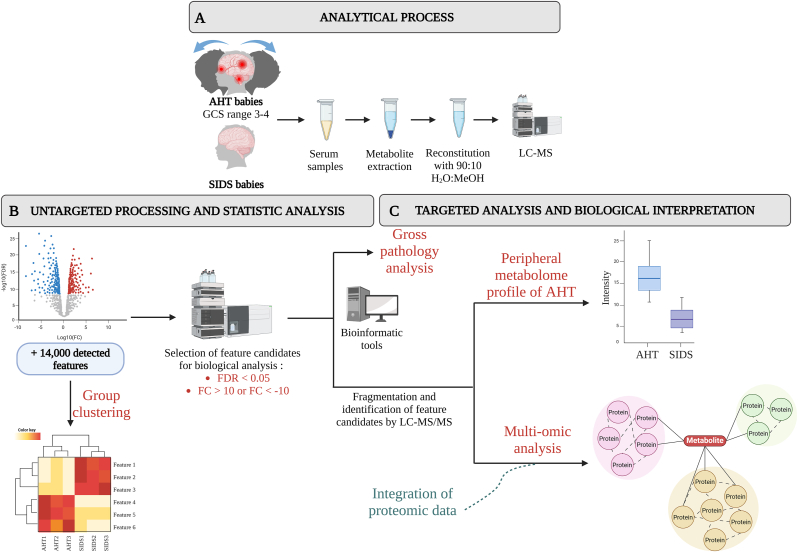
**Approach for metabolomic exploration of AHT peripheral serum**. (A) Serum from AHT and SIDS (group control) cohorts were processed for metabolomic analysis. (B) After filtration with the human metabolome database (HMDB), a total of more than 14,000 features were remaining after filtration. Condition of candidates' selection for targeted metabolomics were set as following: features with FC (AHT cohort against SIDS) > 10 or < −10, FDR <0.05 were kept for fragmentation. (C) More than 1,500 features fulfilled the conditions and were used for enrichment analysis based on features detection. Using bio-informatic tools, identified metabolites showed AHT profile compared to SIDS control group. Also, identified metabolites were integrated with proteomic data for multi-omic analysis.

We analyzed peripheral antemortem blood serum samples of infants with severe AHT (3–4 GCS) and atraumatic controls, infants died of SIDS. With untargeted metabolomics following data processing and cleaning steps, a total of 8,858 and 5,725 features were detected in POS and NEG, respectively (Fig. 1A). The Student's t-test revealed 1,446 features (907 and 539 in POS and NEG, respectively) were significantly different after FDR correction, (q < 0.05) and with 10 times fold-change (FC ≤ −10 and FC ≥ 10) between groups (SIDS versus AHT) (Fig. 1B). This set of 1,446 features underwent targeted metabolomics with fragmentation and identification using LC-MS/MS (Fig. 1C). A total of 53 metabolites were identified using PRM. All identified metabolites, in addition to their molecular characteristics and respective statistical relationships are listed in Supplementary Table 1. Metabolites with increased profile in AHT were evaluated regarding their potential significance to different brain regions. Then, through multi-omic analysis, selected metabolites were correlated with proteomic data. Hereafter, we use the term “metabolite features” to refer to compounds detected via untargeted method, when “metabolites” or “AHT metabolome” refer to the identified compounds from targeted method.

#### 3.1.1 Serum metabolome associates with severe AHT

**Fig. 2:**
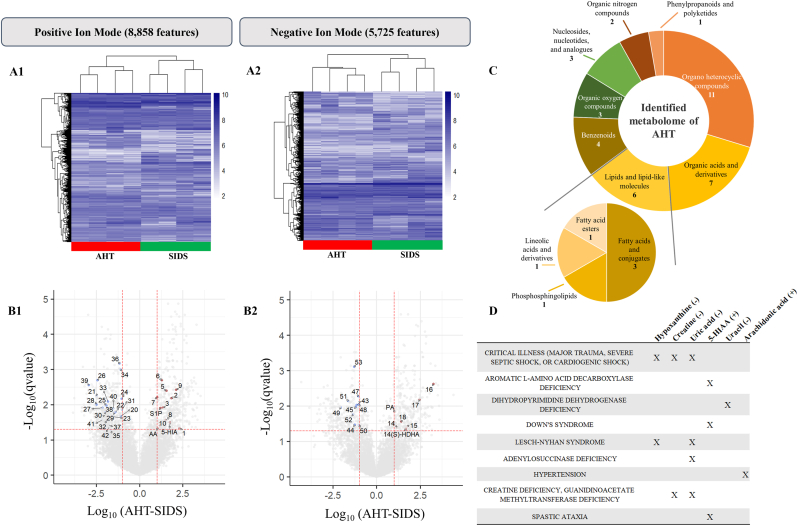
**Overview of peripheral serum metabolome of AHT from untargeted and targeted metabolomic analysis**. Heatmap analysis of feature intensities after data processing and cleaning steps in (A1) POS and (A2) NEG, generated using Euclidean distances. AHT samples were clustered in red and SIDS in green. Volcano plot showing the metabolomics comparison between AHT and SIDS in (B1) POS and (B2) NEG. Indicated metabolites were identified by fragmentation using LC-HRMS/MS technology (Table 1). (C) Chemical composition of the identified metabolome (with assigned HMDB ID) of AHT using ClassyFire categories to classify the metabolite diversity of metabolites. A sub-class of lipids and lipid-like molecules was also assessed. (D) Table of the gross pathologies related to neurological diseases with 6 implicated identified metabolites by LC-HRMS/MS. (+) indicates high intensity level in AHT peripheral blood serum and (−) indicates low intensity level.

To examine the metabolome of both AHT and the control group, we first performed group clustering based on the metabolite feature dataset with known HMDB ID, separately for POS and NEG (Fig. 2A1 and 2A2). Based on 5,725 metabolite features in NEG and 8,858 in POS, both modes demonstrated a statistically significant separation between all samples originating from AHT to the control group.

When comparing AHT infants and the control group at the individual feature level, 907 and 539 metabolite features in POS and NEG, respectively, had significantly different levels (Student's t-test, FDR ≤ 0.05; FC ≤ −10 and FC ≥ 10) between groups (Fig. 2B1 and 2B2). Part of these features, a total of 53 metabolites, were identified with great mzCloud confidence value (from 51 to 99.9 %, Table 1), including 34 decreased metabolites in AHT, while 19 were increased. Metabolites were classified into eight chemical superclasses. Lipids and lipid-like molecules accounted for the third portion of the identified AHT metabolome, all showing an increased profile in peripheral blood of AHT. Lipids were categorized into subclasses: fatty acids and conjugates, phosphosphingolipids, lineolic acids and derivates, and fatty acid esters (Fig. 2C) that reflect their chemical structure and function.

**Table 1:** **Metabolite relative intensities in SIDS and AHT groups are expressed with their peak area log_10_ transformed.** Values are represented as mean ± SD; significant differences between SIDS (n = 4) and AHT (n = 4) are based on Student's t-test (FDR <0.05) and FC > 10 or FC < −10. The identification of metabolites is based on mzCloud Software after fragmentation by LC-HRMS/MS.

	Metabolite	HMDB ID	Chemical structure	Molecular peak (m/z) and ion mode	RT (min)	mzCloud % identification	mzCloud % confidence interval	SIDS (n = 4)	AHT (n = 4)
1.	**Lidocaine**	HMDB0014426	Benzenoids	235.17995 (POS)	3.043	98.4	9.9	5.2278 ± 0.6449	7.5493 ± 0.8865
2.	**Ethyl 3-acetyl-4-oxopentanoate**			187.0961 (POS)	2.499	74.6	65.1	5.0915 ± 0.4138	6.9456 ± 0.1922
3.	**3-Acetyl-2,5-dimethylfuran**	HMDB0029563	Organic oxygen compounds	139.0751 (POS)	4.723	71.1	25	5.486 ± 0.3135	6.8369 ± 0.2463
4.	**Arachidonic acid**	HMDB0001043	Lipids and lipid-like molecules	305.247 (POS)	7.092	70.1	58.5	6.0547 ± 0.2944	7.0979 ± 0.3983
5.	**Isophorone**	HMDB0031195	Organic oxygen compounds	139.1115 (POS)	4.85	64.4	38.6	5.957 ± 0.1881	7.5154 ± 0.2717
6.	**Diethyleneglycol diacetate**			173.0805 (POS)	2.49	58.1	7.9	6.0973 ± 0.152	7.3979 ± 0.1574
7.	**Ethyl sorbate**	HMDB0040463	Lipids and lipid-like molecules	141.0907 (POS)	4.269	55.5	36.9	5.9139 ± 0.1768	6.9376 ± 0.1732
8.	**4-Isobutylbenzoic acid**			179.1064 (POS)	4.141/4.269	53.6/53.8	36.5/36.6	5.8563 ± 0.3976/ 5.5182 ± 0.5728	6.9263 ± 0.1756/ 7.025 ± 0.167
9.	**2-{2-[(2Z)-pent-2-en-1-yl]-3-{[3,4,5-trihydroxy-6-(hydroxymethyl)oxan-2-yl]oxy}cyclopentyl}acetic acid**			397.1828 (POS)	5.554	91.8	9.6	4.9388 ± 0.3129	7.0536 ± 0.3049
10.	**Veratrole**	HMDB0032139	Benzenoids	139.0751 (POS)	3.544	73.3	8.7	5.2624 ± 0.4251	6.4469 ± 0.3123
11.	**5-Hydroxyindole-3-acetic acid**	HMDB0000763	Organo heterocyclic compounds	192.0652 (POS)	4.258	67.3	8.4	4.4126 ± 0.6423	6.1729 ± 0.3535
12.	**D-Erythro-Sphingosine 1-phosphate**	HMDB0000277	Lipids and lipid-like molecules	380.2553 (POS)	6.874	96.5	56.5	6.3145 ± 0.3553	7.6205 ± 0.0588
13.	**Pimelic acid**	HMDB0000857	Lipids and lipid-like molecules	159.0663 (NEG)	3.283	99	95.7	7.3491 ± 0.2646	8.3905 ± 0.1941
14.	**L-(−)-3-Phenyllactic acid**	HMDB0000563	Phenylpropanoids and polyketides	165.0557 (NEG)	3.938	93.2	9.7	5.6534 ± 0.381	6.8175 ± 0.3556
15.	**2-Furoic acid**	HMDB0000617	Organo heterocyclic compounds	111.0087 (NEG)	4.609	78.2	32.3	4.7767 ± 0.3107	6.5576 ± 0.6876
16.	**2-[(2-Chlorobenzyl)thio]-6-methylpyrimidin-4-ol**			265.0196 (NEG)	3.121/3.162	78.2	53	3.7296 ± 0.5858/ 3.5012 ± 0.5	6.7802 ± 0.2326/ 6.7553 ± 0.2603
17.	**BMK glycidic acid**			177.0557 (NEG)	4.414	75.7	8.8	3.6335 ± 0.5805	6.098 ± 0.054
18.	**3-Isopropylmalic acid**	HMDB0012156	Lipids and lipid-like molecules	175.0612 (NEG)	2.97	61.8	8.1	6.0185 ± 0.4489	7.4326 ± 0.3156
19.	**14(S)-HDHA**	HMDB0060044	Lipids and lipid-like molecules	343.2275 (NEG)	6.963/6.972	60.7	54.3	6.9335 ± 0.751	8.6076 ± 0.161
20.	**Hypoxanthine**	HMDB0000157	Organo heterocyclic compounds	137.0455 (POS)	0.985/1.988	99.9	10	6.8022 ± 0.3595	5.7185 ± 0.1628
21.	**Nicotinamide**	HMDB0001406	Organo heterocyclic compounds	123.055 (POS)	0.586	99.9	10	9.2827 ± 0.1489	6.8018 ± 0.562
22.	**Phenacetin**	HMDB0256387	Benzenoids	180.1015 (POS)	1.125	99.3	10	7.0794 ± 0.2395	5.6842 ± 0.3955
23.	**Spermine**	HMDB0001256	Organic nitrogen compounds	203.2226 (POS)	0.311	99.3	95	8.1434 ± 0.4388	6.656 ± 0.333
24.	**Creatine**	HMDB0000064	Organic acids and derivatives	132.0765 (POS)	0.422	98.9	9.9	9.1568 ± 0.1064	8.1181 ± 0.2371
25.	**Caprolactam**	HMDB0062769	Organo heterocyclic compounds	114.0911 (POS)	2.731	98.6	9.9	9.5462 ± 0.5346	7.6264 ± 0.1157
26.	**Uracil**	HMDB0000300	Organo heterocyclic compounds	113.0343 (POS)	0.52	98.1	9.9	8.1453 ± 0.3079	5.7166 ± 0.2668
27.	**Carnosine**	HMDB0000033	Organic acids and derivatives	227.1133 (POS)	0.424	96.8	98.6	7.2707 ± 0.3901	5.1272 ± 0.5
28.	**α-Methylhistamine**	HMDB0243669	–	126.1024 (POS)	0.306	88	35.3	6.0054 ± 0.4851	3.5148 ± 0.5
29.	**2-Naphthylamine**	HMDB0041802	Benzenoids	144.0805 (POS)	2.79	82	9.1	6.6943 ± 0.3739	5.2303 ± 0.3273
30.	**1-Adamantanamine**	HMDB0015051	Organic nitrogen compounds	152.1431 (POS)	4.823	61	8.1	6.3018 ± 0.46	4.2679 ± 0.5882
31.	**2-Aminooctanedioic acid**			190.107 (POS)	3.297	55.3	23	8.0519 ± 0.1159	7.0347 ± 0.297
32.	**Hexanoyl carnitine**	HMDB0000705	–	260.1849 (POS)	3.481	98.2	63.2	9.0437 ± 0.2824	7.1472 ± 0.7028
33.	**Anhydroecgonine**	HMDB0248435	Organo heterocyclic compounds	168.1016 (POS)	0.923	97.2	9.9	7.1414 ± 0.1549	5.3144 ± 0.4533
34.	**Leucyl proline**	HMDB0011175	Organic acids and derivatives	229.1540 (POS)	0.9	96.7	9.8	9.0893 ± 0.1248	7.9883 ± 0.0951
35.	**2′-O-Methylguanosine**	HMDB0001563	Nucleosides, nucleotides, and analogues	298.1140 (POS)	2.07	96.4	9.8	7.6712 ± 0.4258	6.0269 ± 0.7029
36.	**7-Methylguanosine**	HMDB0001563	Nucleosides, nucleotides, and analogues	298.1140 (POS)	1.71	97.4	9.9	7.7948 ± 0.1313	6.6429 ± 0.069
37.	**Uric acid**	HMDB0000289	Organo heterocyclic compounds	169.0354 (POS)	2.057	81	9.1	7.1915 ± 0.2408	5.4131 ± 0.7389
38.	**7-Methylguanine**	HMDB0000897	Organo heterocyclic compounds	166.0721 (POS)	2.069	85.2	33.6	7.1848 ± 0.4218	5.207 ± 0.5137
39.	**N1-Acetylspermine**	HMDB0001186	Organic acids and derivatives	245.233 (POS)	0.304	71.6	41.8	6.5694 ± 0.2233	3.6355 ± 0.5
40.	**N-Methylcaprolactam**			128.1067 (POS)	3.895	61.2	38	7.2669 ± 0.4462	5.7548 ± 0.0946
41.	**1-(2-methoxy-4-nitrophenyl)pyrrolidine**			223.1072 (POS)	1.138	59.1	8	6.3393 ± 0.5415	4.1714 ± 0.6555
42.	**N-cyclooctylurea**			171.1488 (POS)	4.499	51	7.6	8.6283 ± 0.8208	6.8517 ± 0.0366
43.	**DL-Malic acid**	HMDB0000744	–	133.0143 (NEG)	0.472	99.6	88.9	8.645 ± 0.1845	7.5038 ± 0.238
44.	**9-(3-O-Methylpentofuranosyl)-1,9-dihydro-6H-purin-6-one**			281.089 (NEG)	1.99	96.4	9.8	6.8379 ± 0.4185	5.5887 ± 0.3005
45.	**Riboflavin**	HMDB0000244	Organo heterocyclic compounds	375.1307 (NEG)	3.653	93.8	9.7	7.0694 ± 0.1742	5.8772 ± 0.2836
46.	**Xylitol**	HMDB0002917	Organic oxygen compounds	211.0821 (NEG)	0.408	91.7	82.7	7.1989 ± 0.4846	2.5993 ± 0
47.	**Methylmalonic acid**	HMDB0000202	Organic acids and derivatives	117.0193 (NEG)	0.676	90	9.5	9.3417 ± 0.183	8.3041 ± 0.1345
48.	**2-Aminooctanedioic acid**			188.0927 (NEG)	3.296	84.2	56.9	8.6367 ± 0.1203	7.6286 ± 0.2335
49.	**Xanthosine**	HMDB0000299	Nucleosides, nucleotides, and analogues	283.0683 (NEG)	1.077	88.6	9.4	7.9248 ± 0.426	5.881 ± 0.4018
50.	**Capryloyl glycine**	HMDB0000832	Organic acids and derivatives	200.1292 (NEG)	4.364	71.1	8.6	7.3957 ± 0.3991	6.3935 ± 0.13
51.	**α-Aspartylphenylalanine**	HMDB0000706	Organic acids and derivatives	279.0986 (NEG)	2.467	71.2	57.9	7.1093 ± 0.2694	5.4812 ± 0.286
52.	**4-Pyridoxic acid**	HMDB0000017	Organo heterocyclic compounds	182.0458 (NEG)	1.032	60.7	8	8.1534 ± 0.2569	6.8079 ± 0.3659
53.	**N2-Methylguanosine**	HMDB0005862	Nucleosides, nucleotides, and analogues	296.1 (NEG)	1.706	71.2	8.6	7.1098 ± 0.1516	5.8367 ± 0.0571

Next, we analyzed the AHT metabolome in relation to the actual gross pathologies which can be detected in blood. Several gross pathologies related to neurological diseases with the implication of at least one identified metabolite were highlighted (Fig. 2D). The pathologies were: i) critical illness, which is composed of major trauma, severe septic shock or cardiogenic shock (including hypoxanthine, creatine and uric acid), ii) aromatic L-amino acid decarboxylase deficiency (including 5-hydroxyindole-3-acetic acid (5-HIAA)), iii) dihydropyrimidine dehydrogenase deficiency (uracil), iv) down's syndrome (5-HIAA), v) lesh-nyhan syndrome (hypoxanthine and uric acid), vi) adenyloscuccinase deficiency (uric acid), vii) hypertension (arachidonic acid), viii) creatine deficiency/guanidinoacetate methyltransferase deficiency (creatine and uric acid) and ix) spastic ataxia (5-HIAA). Of those metabolites, all were decreased in AHT, except for 5-HIAA and AA which were upregulated. The complete ORA from the feature enrichment can be found in the Supplementary Table 2.

#### 3.1.2 Brain-related metabolites elevated in peripheral bloodstream of AHT

**Fig. 3:**
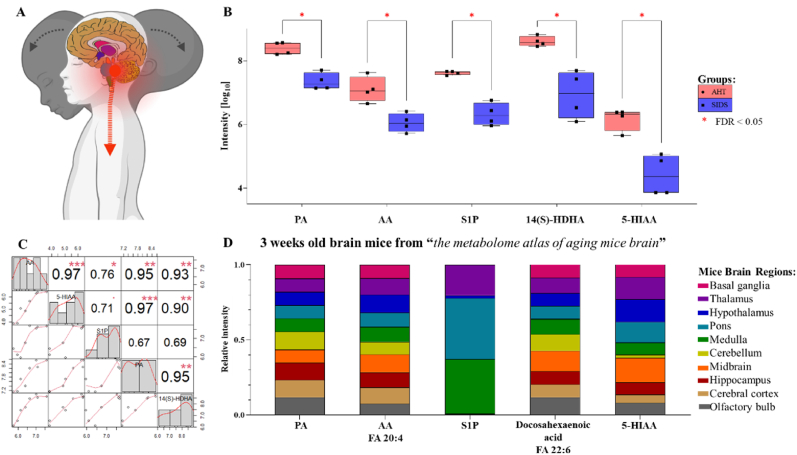
**Focus on five increased metabolites in AHT**. (A) Hypothesis of brain-related metabolites release into the peripheral blood circulation during abuse. (B) Relative intensity of fragmented metabolites expressed by peak area. Columns represent mean ± SD deviation (FDR <0.05 and FC > 10 or FC < −10; AHT, SIDS). Stars ∗ FDR <0.05. (C) Correlation of brain-related metabolites (significant rho = 0.71, df = 6). Stars ∗ >0.8, ∗∗ >0.95, ∗∗∗ >0.97. (D) Relative distribution of the metabolite found in different brain regions of 3 weeks old mice. Reported data of brain specificities from the metabolome atlas of aging mice brain (accessed August 14th, 2023). Legend: 5-hydroxyindole-3-acetic acid (5-HIAA), 14(S)-hydroxy docosahexaenoic acid (14(S)-HDHA), erythro-sphingosine 1-phosphate (S1P), arachidonic acid (AA), pimelic acid (PA).

We further analyzed increased AHT metabolome regarding their potential significance to different brain regions. For that purpose, we used publicly available data of 3 weeks old brain mice from the metabolome atlas of the aging mouse brain (Ding et al., 2021). Metabolites relevance to 10 anatomically defined regions: cerebral cortex (CT), olfactory bulb (OB), hippocampus (HC), hypothalamus (HT), basal ganglia (BG), thalamus (TL), midbrain (MB), pons (PO), medulla (MD), and cerebellum (CB) was assessed. Interestingly, the increased AHT metabolome included four lipids and lipid-like molecules (14(S)-hydroxy docosahexaenoic acid (14(S)-HDHA), erytho-sphingosine 1-phosphate (S1P), arachidonic acid (AA), and pimelic acid (PA)) and one organo heterocyclic compound (5-hydroxyingole-3-acetic acid (5-HIAA)) matching the metabolome database. S1P was predominantly reported in PO and MD brain regions, while the other metabolites were found in all the other regions, although with some slight variations in intensity for some regions that stand at the back of the head (MB, CB, MD, HT, TL, and PO) (Fig. 3B and D). Moreover, 5-HIAA, 14(S)-HDHA, AA, and PA tend to have similar trends across the two-group combined with significant correlation, while S1P showed less significant correlation with the four other metabolites (Fig. 3C). To conclude, these findings support the idea that components from the immature and injured brain could be released into peripheral blood circulation and thus indicate tensions/injuries happening in these susceptible brain regions (Fig. 3A).

### 3.2 Brain-related metabolites associate with protein expression of genes with elevated expression in the brain

**Fig. 4:**
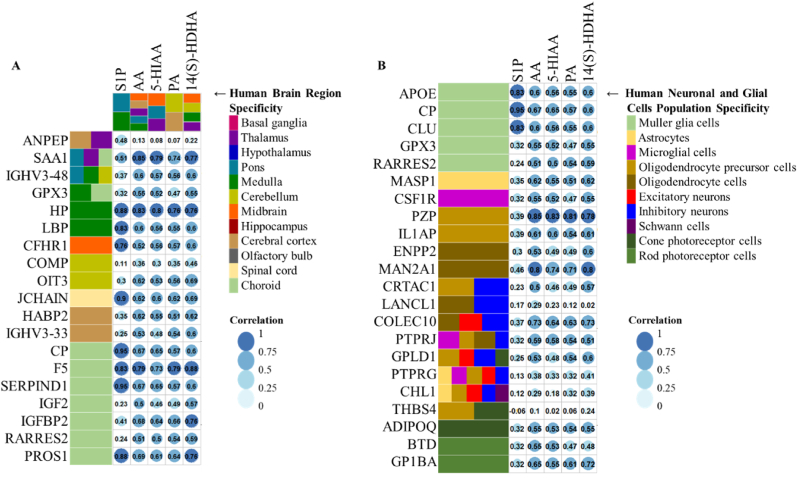
**Multi-omic integration analysis**. (A) Heatmap of Spearman's rank correlation analysis between differential metabolites and proteins with brain region specificities. Brain region specificities were reported from the Human Protein Atlas for proteins and the metabolome atlas of aging mice brain for metabolites. Light blue: poor correlation, dark blue: high correlation. Significant correlations were set at rho = 0.7, df = 6. (B) Heatmap of Spearman's rank correlation analysis between differential metabolites and proteins with glial or neuronal cells specificities. Brain cell specificities were reported from the Human Protein Atlas. Light blue: poor correlation, dark blue: high correlation. Significant correlations were set at rho = 0.7, df = 6. Legend: serum amyloid A-1 protein (SAA1), haptoglobin (HP), lipopolysaccharide-binding protein (LBP), coagulation factor V (F5), heparin cofactor 2 (SERPIND1), vitamin K-dependent protein S (PROS1), ceruloplasmin (CP) and immunoglobulin J chain (JCHAIN), apolipoprotein E (APOE) and clusterin (CLU), pregnancy zone protein (PZP) and alpha-mannosidase 2 (MAN2A1), collectin-10 (COLEC10), platelet glycoprotein Ib alpha chain (GP1BA), aminopeptidase N (ANPEP), immunoglobulin heavy variable 3–48 (IGHV3-48), glutathione peroxidase 3 (GPX3), complement factor H-related protein 1 (CFHR1), cartilage oligomeric matrix protein (COMP), oncoprotein-induced transcript 3 protein (OIT3), hyaluronan-binding protein 2 (HABP2), immunoglobulin heavy variable 3–33 (IGHV3-33), insulin-like growth factor II (IGF2), insulin-like growth factor-binding protein 2 (IGFBP2), retinoic acid receptor responder protein 2 (RARRES2), mannan-binding lectin serine protease 1 (MASP1).

To further confirm the impact of AHT on the brain regions highlighted by the metabolome analysis, we took advantage of the publicly available proteome dataset (Wiskott et al., 2022) to highlight similar tendency for brain compounds to be released into the bloodstream. We focused on the 165 significantly dysregulated proteins and selected proteins with elevated gene expression in the brain using transcriptomic lists from the Human Protein Atlas database at brain regions and brain cells levels (Fig. 4A). Twelve brain regions (CT, OB, HC, HT, BG, TL, MB, PO, MD, CB, spinal cord (SC), and choroid (CD)) and ten brain cells types (muller glia cells (MGC), astrocytes (AST), microglial cells (MCGC), oligodendrocytes precursor cells (OLPC), oligodendrocyte cells (OLC), excitatory neurons (EXN), inhibitory neurons (INN), schwann cells (SCC), cone photoreceptor cells (CPC), and rod photoreceptor cells (RPC)) were assessed. After data processing, 38 proteins were retained (Supplementary Tables 3 and 4), and all of them significantly correlated with at least 1 of the 5 previously highlighted metabolites (5-HIAA, 14(S)-HDHA, AA, PA and S1P).

At brain region level (Fig. 4A), haptoglobin (HP) and coagulation factor V (F5) highly transcribed in MD and CD showed significant correlation with all the 5 metabolites. Serum amyloid A-1 protein (SAA1) from PO, TL and CD regions was significantly correlated with AA, 5-HIAA, PA and 14(S)-HDHA. In addition, five proteins: lipopolysaccharide-binding protein (LBP), complement factor H-related protein 1 (CFHR1), immunoglobulin J chain (JCHAIN), ceruloplasmin (CP) and heparin cofactor 2 (SERPIND1), highly transcribed in MD, MB, SP and CD, respectively, were significantly correlated with S1P metabolite predominantly detected in nearby regions (PO and MD regions) of 3 weeks old mice. At brain cell type level (Fig. 4B), CP, apolipoprotein E (APOE) and clusterin (CLU) proteins, highly transcribed in MGC, revealed significant correlations with S1P metabolite. Pregnancy zone protein (PZP) and alpha-mannosidase 2 (MAN2A1), highly transcribed in OLPC and OLC, respectively, revealed significant correlation with 14(S)-HDHA, PA, AA and 5-HIAA metabolites. Moreover, collectin-10 (COLEC10) protein, highly transcribed in EXN, INC and OLC, showed a slight significant correlation with 14(S)-HDHA and AA metabolites. To conclude, similar variations between proteins and metabolites from specific regions of the brain or from specific brain cell types in peripheral blood serum of AHT provide evidence of tensions/injuries that may happen in brain regions during AHT.

## 4 Discussion

Metabolomic studies of brain trauma have mainly relied on adult acute traumatic brain injury (Orešič et al., 2016; Thomas et al., 2022) and animal models (Anthonymuthu et al., 2019). Here, our metabolomic study on infant serum has shown that circulating metabolomic features are associated with AHT by allowing a clear separation of AHT from SIDS infants. This is an indication of the existence of a specific metabolomic profile for each condition in the child's serum. Specifically, we identified 53 metabolites which showed the most significant changes between groups. The ORA of the AHT metabolome, that was performed in relation to the gross pathologies which can be detected in blood, revealed significant metabolome association to nine neurological diseases (Chong et al., 2018). Creatine deficiency/guanidinoacetate methyltransferase deficiency was highlighted with the implication of decrease of creatine in AHT group. This deficiency is known as an inborn error disease that results in the interruption of creatine formation and its transport, thus disrupting the high energy needed for proper muscle and brain development, causing associated neurological problems (Stöckler et al., 1994). Interestingly, creatine, as well as two other identified metabolites in our study (carnitine and nicotinamide) with decreased levels in AHT, are subject of research in adult TBI for their potential neuroprotective effects allowing better recovery after brain trauma (Roschel et al., 2021; Ainsley et al., 2017; Dolan et al., 2019; Ferreira and McKenna, 2017; Scafidi et al., 2010; Rennie et al., 2015). Furthermore, ORA highlighted the pathology of hypertension with the involvement of AA metabolite. In addition, it is known that subdural hemorrhages in abused baby, documented during medico-legal investigations, could potentially induce hypertension (Geddes et al., 2001; Nickavar and Assadi, 2014). In addition, another metabolite 5-HIAA, has shown a significant implication in hypertension (Chong et al., 2018) due to the involvement of tryptophan on blood pressure regulation (Diaz et al., 2008). Therefore, these findings, in line with the literature (Chevignard and Lind, 2014), reinforce the concern of potential neurological deficits that abused infants may suffer in the long term.

We found that the 6 identified lipids and lipid-like molecules by our study revealed to be all increased in peripheral blood of AHT. This was not surprising since the brain is composed of fifty percent of lipid components (Hamilton et al., 2007) and trauma is known to induce the release of organ-specific molecules into the peripheral blood circulation (Morochovič et al., 2009; Žurek and Fedora, 2012; Arun et al., 2012). With these findings we speculated that increased metabolites found in peripheral blood circulation of AHT originate from the site of brain injury. In order to report the relevance of a given metabolite to a brain region, and since the metabolome atlas of the human brain remains limited (Osetrova et al., 2024; Senko et al., 2024), we used the brain metabolome atlas of 3 weeks old mice (Ding et al., 2021). Thus, we focused on five metabolites (S1P, 14(S)-HDHA, AA, 5-HIAA, and PA) increased in peripheral blood of AHT. Among these metabolites, S1P metabolite showed the most impressive profile, with high specificity in PO and MD brain regions (Ding et al., 2021), which are highly likely to be affected during AHT insults (Choudhary et al., 2014). As described in the literature, S1P is known as a lipid mediator which regulates a wide range of physiological processes, including lymphocyte egress in response to the high levels of S1P in the blood circulation (Schwab et al., 2005; Pappu et al., 2007). Regarding AA and 14(S)-HDHA, they have been reported to account for more than twenty percent of the major fatty acid families found in the brain of mammalians (McNamara and Carlson, 2006; Crawford and Sinclair, 1971; Contreras et al., 2000). More specifically, 14(S)-HDHA is known to be part of the production of maresin-1, resulting in an anti-inflammatory and pro-resolving metabolite (Lu et al., 2010; Dalli and Serhan, 2016; Hong et al., 2014; Dalli et al., 2013). About 5-HIAA, this metabolite is the product of the degradation of serotonin by MAO enzyme in the serotonin pathway (Shajib and Khan, 2015), which is known to control various brain functions, as well as peripheral organs such as the intestine (Gershon, 2012). Since gastrointestinal disease is often attributed to AHT (Jenny et al., 1999) and supported by TBI research (Lu et al., 2009), the shearing of important serotonergic neurons in the brainstem (Mercado et al., 2022), could partially explain gastrointestinal dysfunction affecting abused babies. Although the concentration and the distribution of S1P, 14(S)-HDHA, AA, 5-HIAA, and PA in the human brain remains partially unknown, numerous disease-related metabolomic studies have confirmed the presence of some of these metabolites (Osetrova et al., 2024; Kurano et al., 2024; Qin et al., 2010) or their precursors (Pawlosky and Salem, 2001; Salvan et al., 2023) in the human brain. In addition, metabolic changes at the level of these 5 metabolites are known to play an important role in neuronal disorders for which inflammation, oxidative stress, or neurotransmitter dysregulation are key factors, such as in Alzheimer's disease (Esposito et al., 2008; Kaddurah-Daouk et al., 2011; Ibáñez et al., 2013) and Parkinson's disease (Kaiserova et al., 2021), in Schizophrenia (Senko et al., 2024), TBI (Bohnert et al., 2024) and acute stroke (Kursun et al.). This is why, the detection of these molecules in the peripheral blood circulation of AHT supports the strong dynamic changes occurring in the brain during assault.

Following these interesting findings and to reinforce the potential biological implication of these five metabolites in AHT, we performed integrative analysis correlating metabolomic to proteomic data from their respective samples. For this purpose, we specifically focus on proteins with elevated gene expressions in the brain to highlight significant trends with metabolomics which could explain the release of these components into the peripheral blood. As major results, we were able to show that all five metabolites significantly correlated with HP and F5 proteins, from MD and CD, respectively. In line with our results, the increase of F5 protein in peripheral blood circulation of AHT has already been hypothesized to originate from injured brain regions (Wiskott et al., 2022). In addition, several proteins with elevated gene expression in MD, MB, SC, and CD significantly correlated with S1P, highly elevated in PO and MD brain regions. Despite the small number of biological samples used in our study, the application of metabolomics in AHT seems to be promising. Our data suggest that metabolomic investigation of blood components can become a valuable tool to enhance our understanding of the pathophysiology of AHT and we believe this approach could be integrated into a multi-omics model for AHT screening.

## 5 Conclusion

Here, for the first time, we explored metabolomic changes in peripheral blood circulation of AHT infants. Despite our limited sample size, changes in features from untargeted metabolomic analysis allowed distinct separation of severe AHT infants from SIDS, indicating a selective metabolomic profile. Moreover, we identified a panel of 53 metabolites with significant difference levels between the two groups via targeted metabolomics. Several gross pathologies related to neurological diseases were highlighted, which are in line with long term neurological deficit that might suffer abused infants (Hung, 2020; Mian et al., 2015). Then, by focusing on five increased metabolites (four lipids and lipid-like molecules and one organo heterocyclic compound) in the peripheral blood serum of AHT infants, we speculated a plausible release of these metabolites from the brain regions affected by the shaking mechanism. As some of these metabolites have already been highlighted in different neuronal disorders, the metabolic alterations that we have detected are not exclusive to AHT, but they rather reflect broader patterns of neuronal damage and dysfunction. However, it is remarkable that our multi-omic analysis integrating metabolomic and proteomic data could detect proteins and metabolites coming from similar brain regions in the child's peripheral blood. Confounding changes in the blood metabolome might occur during child development (Finnie and Blumbergs, 2022; Case et al., 2001), or might be influenced by the range and specific type of trauma (Ommaya et al., 2002). Nevertheless, in line with other research demonstrating changes of blood metabolome after brain trauma (Orešič et al., 2016; Anthonymuthu et al., 2019), our integrated analyses highlight a panel of metabolites and proteins significantly altered in severe AHT samples. Although it is currently challenging to determine the precise diagnostic value of each of these biomarkers in AHT, further investigation involving a larger cohort may enhance our understanding of the molecular mechanisms underlying AHT and advance research into its early detection.

## Limitations

The size of the cohort is limited.

## Credit author statement

E.M, W.K, T.S, F.G, S.LG, Y.D, T.F and A.T designed research; E.M, S.LG, T.S and A.T performed research; E.M, S.LG and A.T acquired data; E.M analyzed data and generated figures; E.M drafted the initial manuscript with the contribution of T.S and A.T. All authors critically reviewed and revised the manuscript for important intellectual content.

## Funding/support

This study was supported by Private Foundation Geneva University Hospital.

## Declaration of competing interest

The authors declare no conflicts of interest.

## Data Availability

Metabolomics data are available in Metabolights database with the following ID: MTBLS10778.
