# Supplementary material for: Exploration of circulating metabolites in infants with abusive head trauma
Source: Curr Res Neurobiol. 2025 Jun 28;9:100154. doi: 10.1016/j.crneur.2025.100154 (PMC12280348; doi:10.1016/j.crneur.2025.100154)
Supplement: Multimedia component 1 [file mmc1.docx]

**SUPPLEMENTAL INFORMATION**

**Exploration Of Circulating Metabolites In Infants With Abusive Head Trauma**

Estelle Maret, Tatjana Sajic, Kim Wiskott, Sylvain Le Gludic, Federica Gilardi, Youssef Daali, Tony Fracasso, Aurélien Thomas

**Summary of Supplemental Information**

**Supplementary Figure**

1. **Figure 1.** Principal Component Analysis (PCA).

**Supplementary Tables**

1. **Table 1.** Characteristics of infants and collected samples.
2. **Table 2.** Gross pathologies highlighted by ORA using MetaboAnalyst 5.0.
3. **Table 3.** Brain specificity of proteomic and metabolomic data.
4. **Table 4.** Neuronal and glial cell population specificity of proteomic data.


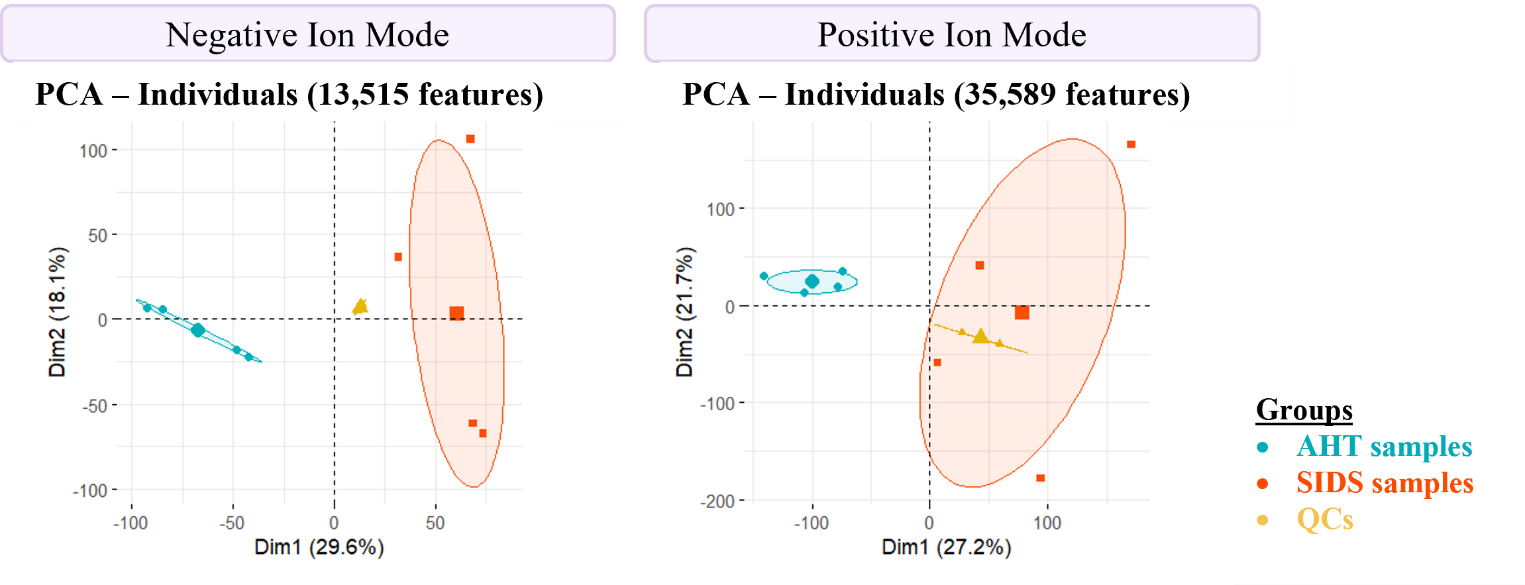


**Supplementary Figure 1 – Principal Component Analysis (PCA).**

PCA scores for NEG and POS ion modes after LC-MS analysis for the first two principal components, comprising 13,515 and 35,589 detected features, respectively.

|  | **Abusive Head Trauma (n=4)**  (AHT, case group^a^) | **Sudden Infant Death (n=4)**  (SIDS, control group) |
| --- | --- | --- |
| **Gender** | | |
| Male | 3 | 4 |
| Female | 1 | 0 |
| **Age** (months) | | |
| Range | 2.5 – 6.8 | 2.9 – 7.5 |
| 0-3 | 1 | 1 |
| 3-6 | 1 | 2 |
| 6-9 | 2 | 1 |
| **Weight** (kg) | | |
| Range (mean) | 5.2 – 8.2 (6.85) | 5.9 – 11 (7.48) |
| **Size** (cm) | | |
| Range (mean) | 59 – 70 (64.25) | 60 – 79 (66) |

**Supplementary Table 1 – Characteristics of infants and collected samples.**

^a^AHT cases display severe TBI (Glasgow Coma Scale (GCS) score range: 3-4).

| Gross pathologies | Total | Hits | P-value | Identified metabolites by our study |
| --- | --- | --- | --- | --- |
| * CRITICAL ILLNESS (MAJOR TRAUMA, SEVERE SEPTIC SHOCK, OR CARDIOGENIC SHOCK) | 6 | 5 | 4.13E-04 | hypoxanthine, creatine, uric acid |
| PURINE NUCLEOSIDE PHOSPHORYLASE DEFICIENCY | 5 | 4 | 0.002 | uric acid |
| * AROMATIC L-AMINO ACID DECARBOXYLASE DEFICIENCY | 12 | 6 | 0.004 | 5-HIAA |
| HYPERBARIC OXYGEN EXPOSURE | 9 | 5 | 0.005 | hypoxanthine, uric acid, arachidonic acid |
| EARLY MARKERS OF MYOCARDIAL INJURY | 14 | 6 | 0.01 | hypoxanthine |
| * DIHYDROPYRIMIDINE DEHYDROGENASE DEFICIENCY | 4 | 3 | 0.01 | uracil |
| * DOWN'S SYNDROME | 4 | 3 | 0.01 | 5-HIAA |
| * 2-HYDROXYGLUTARIC ACIDEMIA (L) | 2 | 2 | 0.02 |  |
| ADDISON'S DISEASE | 2 | 2 | 0.02 |  |
| * GABA TRANSAMINASE DEFICIENCY | 2 | 2 | 0.02 |  |
| XANTHINURIA | 2 | 2 | 0.02 | uric acid |
| * LESCH-NYHAN SYNDROME | 5 | 3 | 0.02 | hypoxanthine, uric acid |
| METABOLITES AFFECTED BY EXERCISE | 5 | 3 | 0.02 | hypoxanthine |
| * ADENYLOSUCCINASE DEFICIENCY | 3 | 2 | 0.06 | uric acid |
| * DOPAMINE BETA-HYDROXYLASE DEFICIENCY | 3 | 2 | 0.06 |  |
| GESTATIONAL DIABETES MELLITUS | 3 | 2 | 0.06 | arachidonic acid |
| * HYPERLYSINEMIA II OR SACCHAROPINURIA | 3 | 2 | 0.06 |  |
| * PHEOCHROMOCYTOMA | 3 | 2 | 0.06 |  |
| SHORT/BRANCHED-CHAIN ACYL-COA DEHYDROGENASE DEFICIENCY | 3 | 2 | 0.06 |  |
| CELIAC DISEASE | 12 | 4 | 0.09 |  |
| * HYPERTENSION | 12 | 4 | 0.09 | arachidonic acid |
| MALNUTRITION | 8 | 3 | 0.1 |  |
| * CREATINE DEFICIENCY, GUANIDINOACETATE METHYLTRANSFERASE DEFICIENCY | 4 | 2 | 0.1 | creatine, uric acid |
| * HEPATIC ENCEPHALOPATHY | 4 | 2 | 0.1 |  |
| * HYPERORNITHINEMIA WITH GYRATE ATROPHY (HOGA) | 4 | 2 | 0.1 |  |
| RHABDOMYOLYSIS | 4 | 2 | 0.1 | creatine |
| * SPONTANEOUS RECURRENCE OF METHAMPHETAMINE (MAP)-INDUCED PSYCHOSIS | 4 | 2 | 0.1 |  |
| * ISOVALERIC ACIDEMIA | 9 | 3 | 0.1 |  |
| BREAST CANCER | 5 | 2 | 0.1 | spermine |
| FRUCTOSE-1,6-DIPHOSPHATASE DEFICIENCY | 5 | 2 | 0.1 | uric acid |
| N-ACETYLGLUTAMATE SYNTHETASE DEFICIENCY. NAGS DEFICIENCY | 5 | 2 | 0.1 |  |
| * SPASTIC ATAXIA | 5 | 2 | 0.1 | 5-HIAA |

**Supplementary Table 2 – Gross pathologies highlighted by ORA using MetaboAnalyst 5.0.**

1,446 features detected by our analysis and showing FC ≤ -10 or FC ≥ 10, Student’s t-test, FDR ≤ 0.05 were enriched using HMDB leading to 1,659 potential metabolites. ORA was performed with an enriched number of features but restricted to 440 metabolites available in MetaboAnalyst 5.0. Stars (*) indicate neurology gross pathologies. The total column includes the number of metabolites registered in MetaboAnalyst database implicated into the corresponding gross pathology. The hits column indicates the number of our features that are implicated in the corresponding gross pathology. P-value was performed by MetaboAnalyst analysis.

|  | **Pons** | **Medulla** | **Midbrain** | **Cerebellum** | **Cerebral Cortex** | **Thalamus** | **Choroid** | **Spinal Cord** |
| --- | --- | --- | --- | --- | --- | --- | --- | --- |
| FA 20:4 (AA)  (Intensity 10^8^) | ++ | ++ | ++++ | + | +++ | +++ |  |  |
| 5-HIAA  (Intensity 10^2^) | +++ | ++ | ++++ | + | ++ | +++ |  |  |
| S1P  (Intensity 10^5^) | ++++ | ++++ | + | + | + | +++ |  |  |
| Pimelic acid  (Intensity 10^6^) | ++ | ++ | ++ | ++++ | +++ | ++ |  |  |
| FA 22:6 (14(S)-HDHA)  (Intensity 10^7^) | + | ++ | ++++ | +++ | + | ++ |  |  |
| SAA1 | + |  |  |  |  | + | + |  |
| IGHV3-48 | + | + |  | + |  |  |  |  |
| GPX3 |  | + |  |  |  |  | + |  |
| HP |  | + |  |  |  |  |  |  |
| LBP |  | + |  |  |  |  |  |  |
| CFHR1 |  |  | + |  |  |  |  |  |
| COMP |  |  |  | + |  |  |  |  |
| OIT3 |  |  |  | + |  |  |  |  |
| JCHAIN |  |  |  |  |  |  |  | + |
| ANPEP |  |  |  |  | + | + |  |  |
| HABP2 |  |  |  |  | + |  |  |  |
| IGHV3-33 |  |  |  |  | + |  |  |  |
| CP |  |  |  |  |  |  | + |  |
| F5 |  |  |  |  |  |  | + |  |
| SERPIND1 |  |  |  |  |  |  | + |  |
| IGF2 |  |  |  |  |  |  | + |  |
| IGFBP2 |  |  |  |  |  |  | + |  |
| RARRES2 |  |  |  |  |  |  | + |  |
| PROS1 |  |  |  |  |  |  | + |  |

**Supplementary Table 3 – Brain specificity of proteomic and metabolomic data.**

Brain specificities of proteomic and metabolomic data were established by transcriptome reported from the Human Protein Atlas, and the metabolome atlas of the aging mouse brain, respectively (accessed August 14^th^, 2023).

|  | *Muller glia cells* | *Astrocytes* | *Microglia cells* | *Oligodendrocyte precursor cells* | *Oligodendrocyte cells* | *Excitatory cells* | *Inhibitory cells* | *Schwann cells* | *Cone photoreceptor cells* | *Rod photoreceptor cells* |
| --- | --- | --- | --- | --- | --- | --- | --- | --- | --- | --- |
| APOE | X |  |  |  |  |  |  |  |  |  |
| CP | X |  |  |  |  |  |  |  |  |  |
| CLU | X |  |  |  |  |  |  |  |  |  |
| GPX3 | X |  |  |  |  |  |  |  |  |  |
| RARRES2 | X |  |  |  |  |  |  |  |  |  |
| MASP1 |  | X |  |  |  |  |  |  |  |  |
| CSF1R |  |  | X |  |  |  |  |  |  |  |
| PZP |  |  |  | X |  |  |  |  |  |  |
| IL1RAP |  |  |  | X |  |  |  |  |  |  |
| ENPP2 |  |  |  |  | X |  |  |  |  |  |
| MAN2A1 |  |  |  |  | X |  |  |  |  |  |
| CRTAC1 |  |  |  | X |  |  | X |  |  |  |
| LANCL1 |  |  |  |  | X |  | X |  |  |  |
| COLEC10 |  |  |  |  | X | X | X |  |  |  |
| PTPRJ |  |  | X | X | X |  | X |  |  |  |
| GPLD1 |  |  |  | X |  | X | X |  | X |  |
| PTPRG |  | X | X | X |  | X | X |  |  |  |
| CHL1 |  | X |  | X |  | X | X | X |  |  |
| THBS4 |  |  |  | X |  |  |  |  | X |  |
| ADIPOQ |  |  |  |  |  |  |  |  | X |  |
| BTD |  |  |  |  |  |  |  |  |  | X |
| GP1BA |  |  |  |  |  |  |  |  |  | X |

**Supplementary Table 4 – Neuronal and glial cell population specificity of proteomic data.**

Neuronal and glial cell specificities of proteomic data were established by transcriptome reported from The Human Protein Atlas (accessed August 14^th^, 2023).
